# Mechanism of Cross-Resistance to Fusion Inhibitors Conferred by the K394R Mutation in Respiratory Syncytial Virus Fusion Protein

**DOI:** 10.1128/JVI.01205-21

**Published:** 2021-09-27

**Authors:** Wei Tang, Yueyue Li, Qiaoyun Song, Ziqin Wang, Manmei Li, Qiwei Zhang, Ying Wang, Wencai Ye, Yaolan Li

**Affiliations:** a Center for Bioactive Natural Molecules and Innovative Drugs Research, College of Pharmacy, Jinan University, Guangzhou, China; b Guangdong Province Key Laboratory of Pharmacodynamic Constituents of TCM & New Drugs Research, Jinan University, Guangzhou, China; c Guangdong Provincial Key Laboratory of Virology, Institute of Medical Microbiology, Jinan University, Guangzhou, China; University of Kentucky College of Medicine

**Keywords:** fusion glycoprotein, K394R mutation, fusion inhibitor, membrane fusion, cross-resistance

## Abstract

The fusion glycoprotein (F) is essential for respiratory syncytial virus (RSV) entry and has become an attractive target for anti-RSV drug development. Despite the promising prospect of RSV F inhibitors, issues of drug resistance remain challenging. In this study, we established a dual-luciferase protocol for RSV fusion inhibitor discovery. A small-molecule inhibitor, salvianolic acid R (LF-6), was identified to inhibit virus-cell and cell-cell fusion mediated by the RSV F protein. Sequence analysis of the resultant resistant viruses identified a K394R mutation in the viral F protein. The K394R mutant virus also conferred cross-resistance to multiple RSV fusion inhibitors, including several inhibitors undergoing clinical trials. Our study further showed that K394R mutation not only increased the triggering rate of F protein in prefusion conformation but also enhanced the fusion activity of F protein, both of which were positively correlated with resistance to fusion inhibitors. Moreover, the K394R mutation also showed cooperative effects with other escape mutations to increase the fusion activity of F protein. By substitution of K394 into different amino acids, we found that K394R or K394H substitution resulted in hyperfusiogenic F proteins, whereas F variants with other substitutions exhibited less fusion activity. Both K394R and K394H in F protein exhibited cross-resistance to RSV fusion inhibitors. Collectively, these findings reveal a positive correlation between the membrane fusion activity of F protein and the resistance of corresponding inhibitors. All of the results demonstrate that K394R in F protein confers cross-resistance to fusion inhibitors through destabilizing F protein and increasing its membrane fusion activity.

**IMPORTANCE** Respiratory syncytial virus (RSV) causes serious respiratory tract disease in children and the elderly. Therapeutics against RSV infection are urgently needed. This study reports the discovery of a small-molecule inhibitor of RSV fusion glycoprotein by using a dual-luciferase protocol. The escape mutation (K394R) of this compound also confers cross-resistance to multiple RSV fusion inhibitors that have been reported previously, including two candidates currently in clinical development. The combination of K394R with other escape mutations can increase the resistance of F protein to these inhibitors through destabilizing F protein and enhancing the membrane fusion activity of F protein. By amino acid deletion or substitution, we found that a positively charged residue at the 394th site is crucial for the fusion ability of F protein, as well as for the cross-resistance against RSV fusion inhibitors. These results reveal the mechanism of cross-resistance conferred by the K394R mutation and the possible cross-resistance risk of RSV fusion inhibitors.

## INTRODUCTION

Respiratory syncytial virus (RSV) is a major pathogen of acute lower respiratory infection that leads to bronchiolitis and pneumonia diseases in children, as well as in the elderly population (1, 2). Worldwide, RSV is estimated to cause more than 3 million hospitalizations and nearly 66,000 deaths annually in children under 5 years of age (3, 4). In the United States, RSV leads to 177,000 hospitalizations and 14,000 deaths annually among adults over the age of 65 (2). Despite great efforts that have been advanced in past decades, a safe and effective vaccine for preventing RSV infection remains unavailable. Therapeutic drugs currently approved against RSV infection are limited to ribavirin and palivizumab. Ribavirin is a nucleoside analogue with broad-spectrum antiviral activities and is associated with limitations, such as severe adverse effects and insufficient efficacy *in vivo* (5). Passive immunoprophylaxis with palivizumab (Synagis), a humanized monoclonal antibody against RSV fusion (F) protein, is the only approved intervention for the prevention of RSV infection, but its use is restricted due to the high cost of treatment and inevitable monthly repeat injections (6). Therefore, the development of alternative anti-RSV drugs is urgently needed.

The attachment glycoprotein (G) and fusion glycoprotein (F) are two major surface glycoproteins of RSV that play important roles during virus entry (7, 8). The RSV F gene is more conserved than the RSV G gene (9). Moreover, recombinant RSV lacking the G protein can still infect host cells and produce progeny virions (10, 11); in contrast, deletion of the F protein leads to the loss of virus entry ability (12), suggesting that the F protein is indispensable for RSV infection. The F protein is initially expressed as an inactive precursor, F0 (13). In the Golgi apparatus, F0 is cleaved at the N and C termini of pep27 by a furin-like protease, generating two disulfide-linked subunits, N-terminal polypeptide (F2) and C-terminal transmembrane polypeptide (F1) (14), which form a trimer of F2-F1 heterodimers in the metastable prefusion state (15). Following the viral attachment, prefusion F undergoes a dramatic conformational change and inserts its fusion peptide into the host cell membrane (16). The rearrangement of metastable prefusion F into extremely stable postfusion F leads to virus-cell fusion and cell-cell fusion.

The search for fusion inhibitors has become an attractive strategy for anti-RSV drug development. Most of the RSV fusion inhibitors that have been reported recently act as antagonists of RSV F rearrangement (17–21). These inhibitors can bind to a 3-fold-symmetric pocket adjacent to fusion peptide in the central cavity of prefusion F trimer, thereby blocking the conformational change of prefusion F into postfusion F (22). Some of these inhibitors exhibit potent *in vitro* and *in vivo* anti-RSV activity, such as BMS-433771, TMC-353121, JNJ-2408068, JNJ-53718678, GS-5806, and AK-0529 (17–19, 23, 24). In particular, JNJ-53718678, GS-5806, and AK-0529 have entered clinical trials (ClinicalTrials registration no. NCT04056611, NCT02135614, and NCT04231968). All of these inhibitors can elicit resistant mutants *in vitro* or *in vivo*. As previously reported, resistance mutations for RSV fusion inhibitors cluster around a central cavity adjacent to fusion peptide in prefusion F trimer (22). They mediate viral resistance through direct or indirect mechanisms (22, 25). The direct mechanisms include mutations at the residues that directly contact the inhibitors (e.g., F488L) or mutations at the residues that hinder the rearrangement of prefusion F required to accommodate inhibitor binding (e.g., D489Y) (22). The indirect mechanisms involve altering the stability and triggering rate of F protein (e.g., D401E and D489E), which results in a narrower window of opportunity for inhibitors to bind (25). Previous studies have shown that K394R is an escape mutation for several RSV fusion inhibitors, such as TMC-353121 and BMS-433771 (18, 26). The RSV variant harboring a K394R/S398L double mutation in the F protein confers more than 30,000-fold resistance to TMC-353121 compared to that of the wild-type virus, whereas a S398L or D486N single mutation in the viral F protein only results in 194-fold or 2,474-fold resistance to this inhibitor, respectively (18). The K394R single mutation in F protein causes 1,250-fold resistance to BMS-433771 (26). These studies reveal that K394R mutation is crucial for viral resistance; however, whether it confers cross-resistance to other structural types of RSV fusion inhibitors, especially to the inhibitors in clinical development, and its cross-resistance mechanism remain unclear.

Here, we established a dual-luciferase protocol for viral fusion inhibitor discovery and identified salvianolic acid R (LF-6) as a new RSV fusion inhibitor. Salvianolic acid R is a natural compound isolated from Mesona chinensis Benth., an herbaceous plant that is widely distributed in tropical and subtropical regions. This compound suppressed RSV infection by blocking virus-cell and cell-cell fusion. The escape variants for LF-6 contain a K394R mutation in the viral F protein. The viral variant harboring the K394R mutation also conferred cross-resistance to RSV fusion inhibitors with a diversity of structures. Furthermore, the K394R mutation or its combination with other escape mutations increased the triggering rate and the membrane fusion activity of F protein, both of which were positively correlated with the cross-resistance of F protein against these inhibitors. The *in vitro* fitness of K394R mutant virus was also evaluated in this study. Our findings demonstrate the potential resistance risk in RSV F-targeted drug development and provide new insight into K394R-mediated cross-resistance.

## RESULTS

### Identification of LF-6 as an inhibitor of cell-cell fusion mediated by RSV F protein.

**FIG 1:**
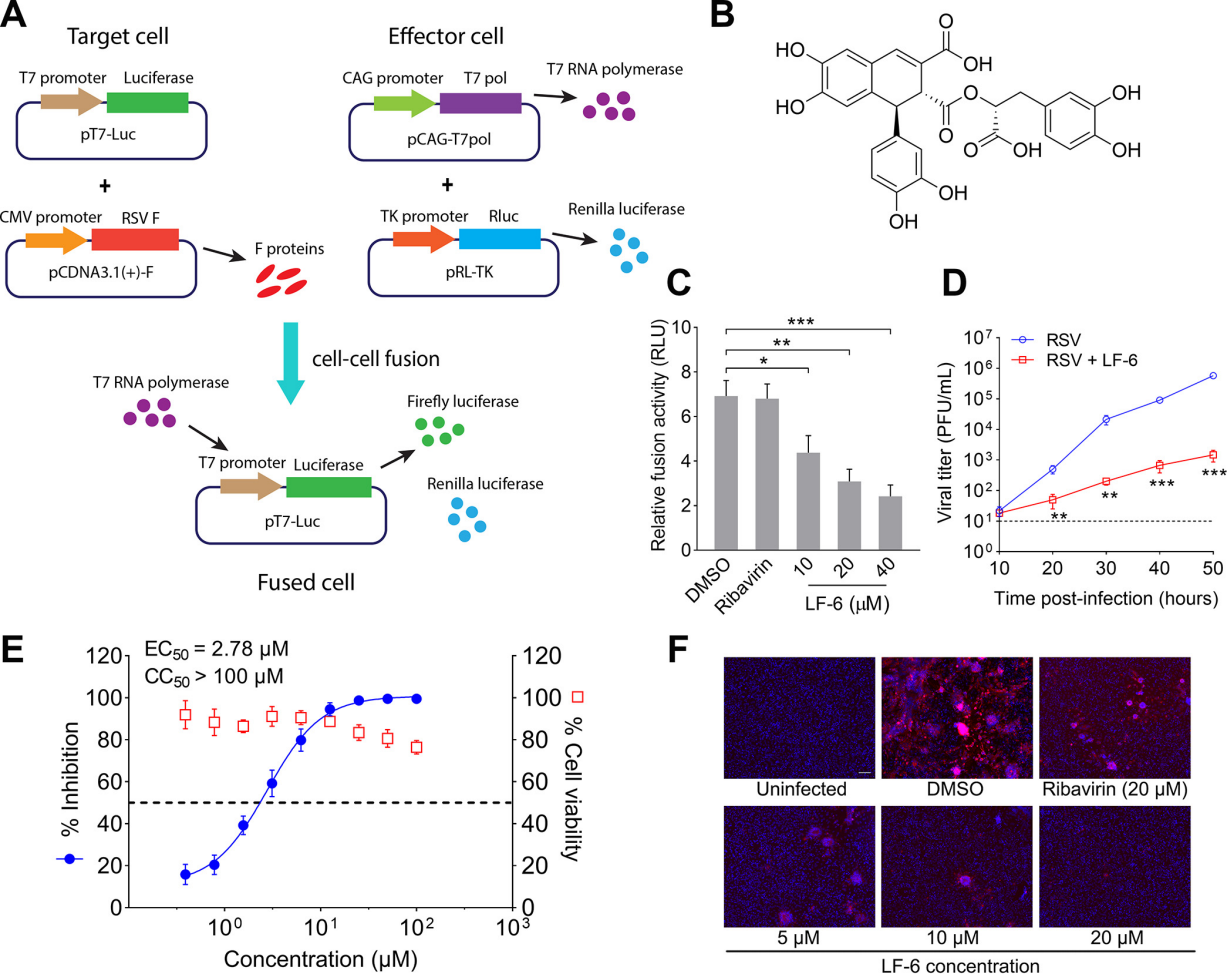
Identification of LF-6 as a cell-cell fusion inhibitor against respiratory syncytial virus (RSV). (A) Schematic representation of a dual-luciferase reporter model for screening of inhibitors capable of blocking cell-cell fusion mediated by RSV fusion glycoprotein (F). (B) Chemical structure of LF-6. (C) Quantitative cell-cell fusion activity determined by dual-luciferase reporter assay. The activities of firefly luciferase and *Renilla* luciferase were measured after transfection of RSV F plasmids in the presence or absence of LF-6. Values are normalized to *Renilla* luciferase activity and shown as mean ± standard deviation (SD); *n *=* *3 biological replicates. *, *P < *0.05; **, *P < *0.01; ***, *P < *0.001. (D) Growth of RSV in HEp-2 cells that were treated with LF-6 (20 μM). Data represent the mean ± SD; *n *=* *3 biological replicates. **, *P < *0.01; ***, *P < *0.001. (E) Effects of LF-6 on cell viability and virus yields as determined by plaque assay. Values were normalized to dimethyl sulfoxide (DMSO)-treated cells and shown as mean ± SD; *n *=* *3 biological replicates. (F) Expression level of F protein in RSV-infected cells in the presence of ribavirin or LF-6 at different concentrations. Bar, 100 μm.

As a major part of our work over the past decade, we have constructed an in-house compound library composed of more than 2,000 natural small molecules that were isolated from about 100 species of medicinal plants. Some of the compounds from this library exhibit considerable antiviral activities (27–30). In this study, an optimized dual-luciferase reporter assay was conducted to investigate the capacity of the compounds with anti-RSV activities to inhibit cell-cell fusion mediated by the RSV F protein (Fig. 1A). The target cells in the assay were transiently cotransfected with pcDNA3.1(+) expressing the full length of the RSV F gene and pT7-Luc plasmids encoding firefly luciferase under the control of the T7 promoter. The effector cells were transiently cotransfected with pRL-TK plasmids containing Renilla luciferase gene and pCAG-T7 Pol plasmids expressing T7 RNA polymerase. Following cell-cell fusion between target cells and effector cells, T7 RNA polymerase in effector cells was spread to the larger fused cell and initiated the expression of firefly luciferase, which correlated with the cell-cell fusion activity.

We found that several salvianolic acid derivatives isolated from M. chinensis were capable of suppressing the cell-cell fusion mediated by RSV F protein. In particular, salvianolic acid R (LF-6) significantly inhibited the cell-cell fusion in a concentration-dependent manner (Fig. 1B and C), whereas ribavirin, as expected, did not affect this process. Next, three sets of experiments were carried out to determine the inhibitory effect of LF-6 on RSV infection. First, viral growth kinetics in RSV-infected cells were assessed at indicated intervals postinfection. At 50 h postinfection, a reduction of about 400-fold in viral growth was observed in cells that were treated with LF-6 (Fig. 1D). Second, virus titers in cells that were treated with increasing concentrations of LF-6 were measured by virus yield assay. LF-6 suppressed the production of progeny virions at these concentrations without exhibiting substantial cytotoxicity (Fig. 1E). Third, immunofluorescent staining results indicated that the viral protein levels in RSV-infected cells were substantially reduced in the presence of LF-6 at concentrations higher than 5 μM (Fig. 1F). In contrast, the viral protein levels in cells that were treated with 20 μM ribavirin were still maintained at a high level. Collectively, these results suggest that the reduction of virus replication is correlated with the suppression of cell-cell fusion by LF-6.

### LF-6 inhibits the virus-cell fusion without disturbing the viral attachment.

**FIG 2:**
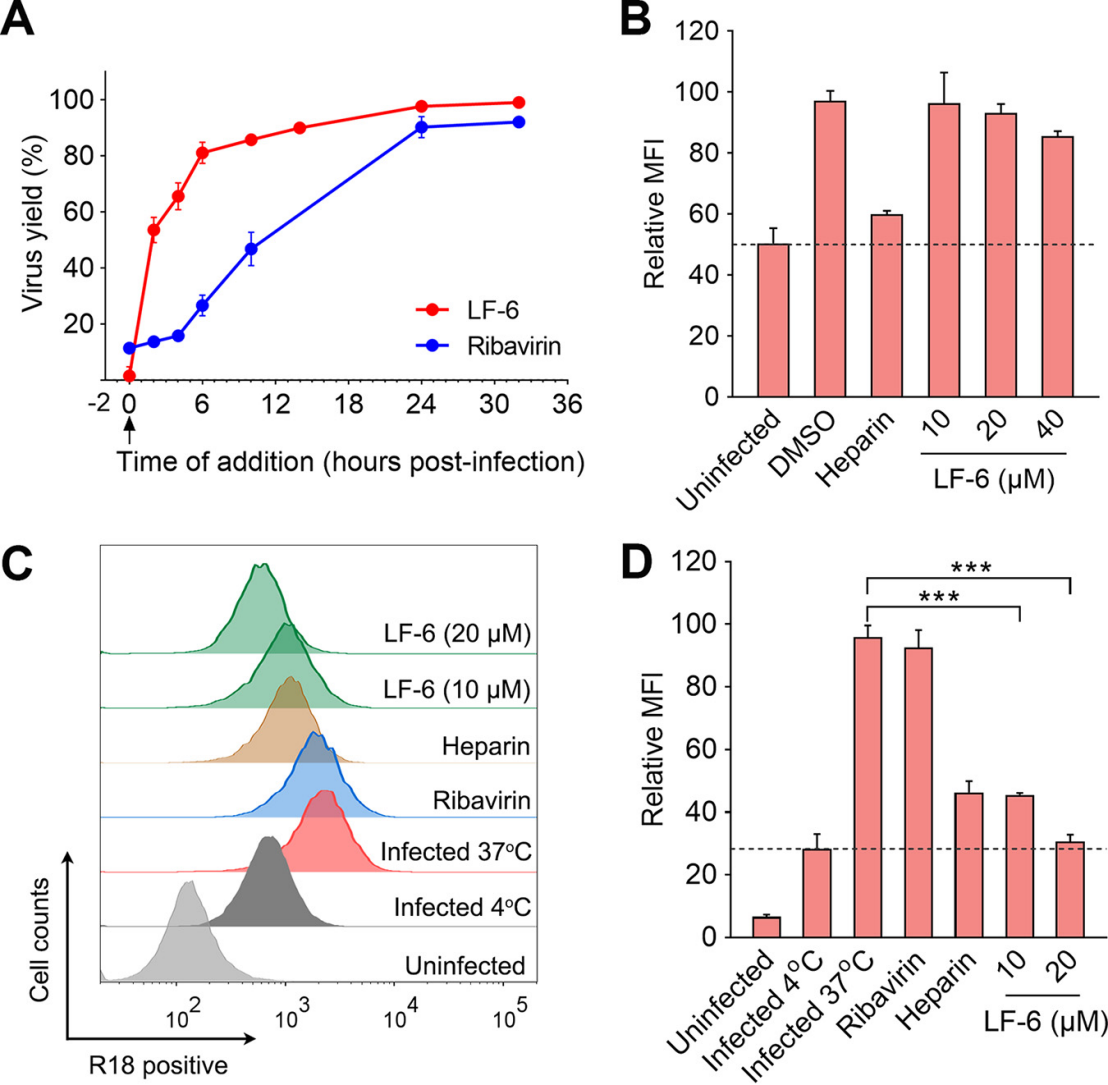
Inhibitory effect of LF-6 on virus-cell fusion. (A) A time-of-addition assay was performed to investigate which stage in the viral life cycle was inhibited by LF-6. HEp-2 cells were infected with RSV and treated with LF-6 (10 μM) or ribavirin (10 μM) at indicated time points after exposure of cells to the virus. Virus yields in the cells were assessed by plaque assay. Data are mean ± SD; *n *=* *3 biological replicates. (B) No significant inhibition of viral attachment was observed in LF-6-treated cells. HEp-2 cells were infected with RSV in the presence or absence of LF-6 or heparin (4 μM). After infection for 1 h at 4°C, cells were incubated with anti-RSV monoclonal antibody and Alexa Fluor 488-conjugated secondary antibody. Mean fluorescence intensity (MFI) correlated with the amount of RSV on the cell surface was quantified by flow cytometry. Data are mean ± SD; *n *=* *3 biological replicates. (C, D) Inhibitory effects of LF-6 on the fusion process of octadecyl rhodamine B (R18)-labeled RSV with the cell membrane. HEp-2 cells were infected with RSV-R18 in the presence of LF-6 (10 μM or 20 μM), ribavirin (20 μM), or heparin (4 μM). After infection for 2 h at 37°C, R18-associated MFI was determined by flow cytometry and analyzed using FlowJo v.10.0. Data are mean ± SD; *n *=* *3 biological replicates. ***, *P < *0.001.

A time-of-addition assay was employed to determine which stage in the viral life cycle was inhibited by LF-6. In this assay, LF-6 was added to cells at different time points after exposure of the cells to RSV. Viral titers in cells were measured at 40 h postinfection. Our results indicated that virus yield was potently suppressed by treatment with LF-6 at 0 h or 2 h after the viral infection, whereas the anti-RSV activity of LF-6 was dramatically attenuated when the compound was added to cells later than 4 h after infection (Fig. 2A). These results suggest that LF-6 inhibits RSV infection, presumably by blocking the virus entry into cells.

During entry of RSV into cells, the virions are first adsorbed on the cell surface and then initiate virus-cell fusion. To assess which step during virus entry was blocked by LF-6, viral attachment and viral fusion assays, respectively, were performed. In the viral attachment assay, cells were treated with LF-6 and then infected with RSV at 4°C to allow the virus to associate with the cell membrane but prevent membrane fusion. The adsorbed virions on the cell surface were stained with specific anti-RSV F antibodies and quantified using flow cytometry. Our results demonstrated that the abundance of RSV on the cell membrane was not affected by the treatment of LF-6 even at a concentration of 14-fold greater than the the concentration of inhibitor that reduced 50% of the cytopathic effect (CPE) (EC_50_), whereas it was significantly decreased when the cells were pretreated with heparin, a broad-spectrum viral attachment inhibitor (Fig. 2B). These results indicate that attachment of RSV with the cell membrane was not inhibited by LF-6.

To determine whether virus-cell fusion was inhibited by LF-6, RSV virions were labeled with a lipophilic probe, octadecyl rhodamine B (R18), which is self-quenched when presented on the surface of virions at a high concentration. Following the virus-cell fusion at 37°C, the R18 probes are diffused into the cell membrane, which eliminates self-quenching between the probes. As a result, the fluorescence intensity emitted by R18 was increased remarkably and can be detected by flow cytometry. Our results demonstrated that R18-associated fluorescence in cells was maintained at an extremely low level when infection was performed at 4°C, whereas it was dramatically increased after infection for 2 h at 37°C (Fig. 2C and D). When infected at 37°C, the fluorescence intensity in the cells that were treated with LF-6 was significantly decreased compared to that in untreated cells, suggesting that diffusion of R18 on virions to the cell membrane was blocked by LF-6. As expected, the negative control, ribavirin, did not disrupt the diffusion of R18. Taken together, these results suggest that virus-cell fusion was inhibited by LF-6.

### LF-6 inhibits the early stage of viral fusion with the cell membrane.

**FIG 3:**
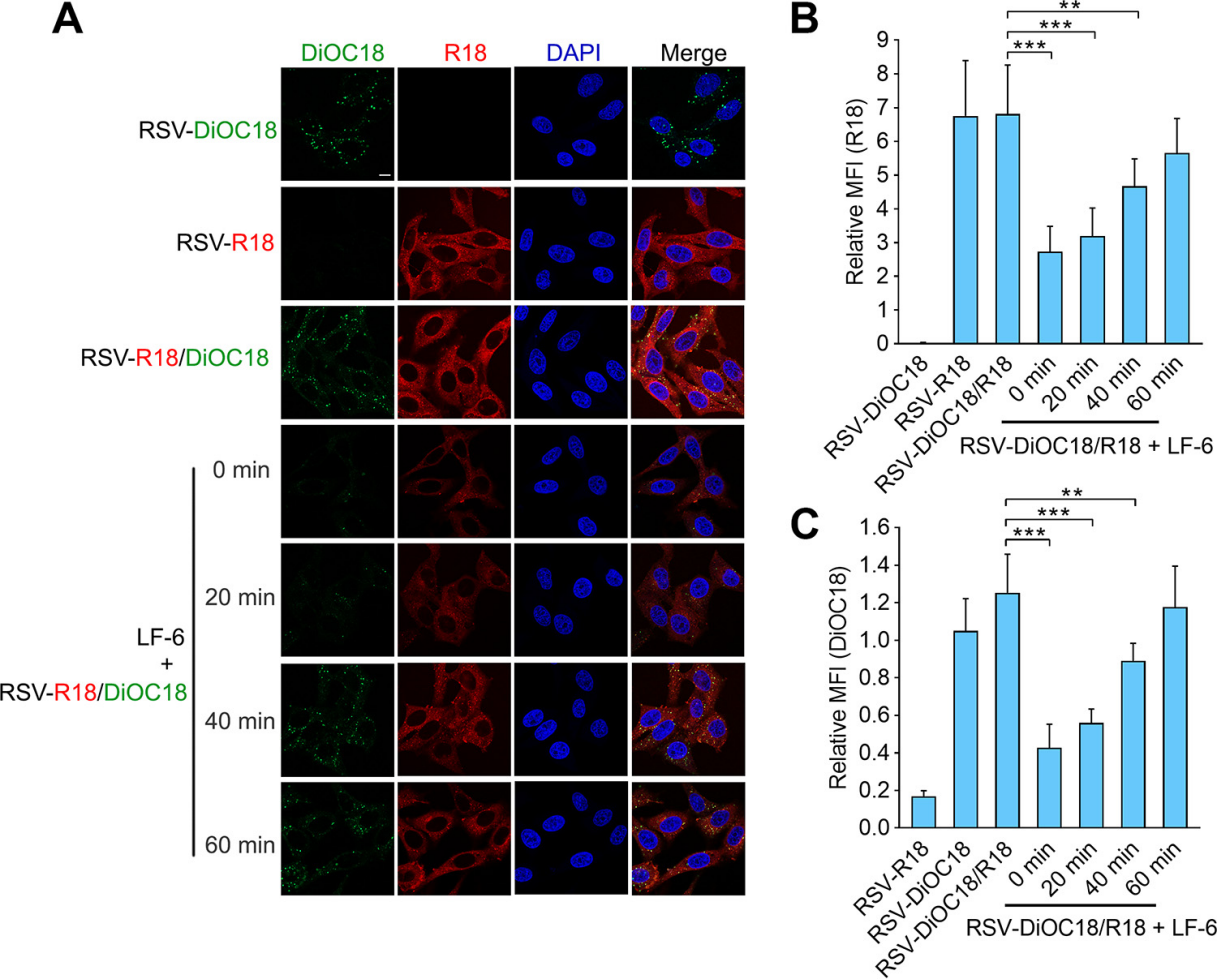
LF-6 inhibits the early stage of virus-cell fusion. (A) Diffusion of R18 and 3,3′-dioctadecyloxacarbocyanine perchlorate (DiOC_18_) probes from RSV surface to cell membrane in the presence of LF-6. HEp-2 cells were infected with R18-, DiOC_18_-, or R18/DiOC_18_-labeled virus and treated with LF-6 (20 μM) at different time points after viral infection. Two hours after the viral infection at 37°C, cells were stained with 4′,6-diamidino-2-phenylindole (DAPI) and subjected to a confocal laser for detecting R18 and DiOC_18_-associated fluorescence. Bar, 10 μm. (B, C) The R18-associated (B) or DiOC_18_-associated (C) fluorescence intensities were quantified and analyzed using ZEN imaging software. Data are mean ± SD; *n *=* *3 biological replicates. **, *P < *0.01; ***, *P < *0.001.

To investigate which step in the virus-cell fusion was blocked by LF-6, the virions were labeled with two lipophilic probes, octadecyl rhodamine B (R18) and 3,3′-dioctadecyloxacarbocyanine perchlorate (DiOC_18_). When presented on the viral membrane, DiOC_18_-associated fluorescence (Fig. 3A, as shown in green) was self-quenched and undergo fluorescence resonance energy transfer (FRET) to R18 probes (Fig. 3A, shown in red), thereby increasing the R18-associated fluorescence intensity. Following the virus-cell fusion at 37°C, both R18 and DiOC_18_ probes on the surface of RSV virions were diffused into the target cell membrane, resulting in the release of self-quenching and elimination of FRET. As a result, DiOC_18_-associated green fluorescence was dramatically enhanced following the virus-cell fusion. In this assay, cells were treated with LF-6 at 0, 20, 40, and 60 min postinfection. DiOC_18_- and R18-associated fluorescence was observed at a high level after the virus-cell fusion, which was potently suppressed in LF-6-treated cells (Fig. 3A to C). In particular, the fluorescence emitted by DiOC_18_ was maintained at an extremely low level when LF-6 was added to the cells at 0 or 20 min postinfection compared to that in untreated cells, suggesting that virus-cell fusion was blocked by LF-6. However, the inhibitory effect was markedly attenuated when LF-6 was added to RSV-infected cells at 60 min postinfection, suggesting that LF-6 inhibits the early stage of virus-cell fusion.

### Escape mutations conferring resistance to LF-6 are located at the RSV F protein.

To identify the potential target of LF-6 against RSV, RSV strain A2 was grown in HEp-2 cells in the presence or absence (parallel control) of LF-6. The viral variants conferring resistance to LF-6 were generated after continuous culturing for 9 passages with treatment by increasing concentrations of LF-6. The EC_50_ of LF-6 against the resistant virus was increased more than 71-fold compared to that of the parallel control virus. Full-length viral genomes were divided into various segments and amplified by PCR. Sequence analysis of these segments indicated that 4 of the 6 isolates of the resistant viruses had a K394R mutation in the viral F gene, whereas 2 isolates had an additional V207M mutation.

**FIG 4:**
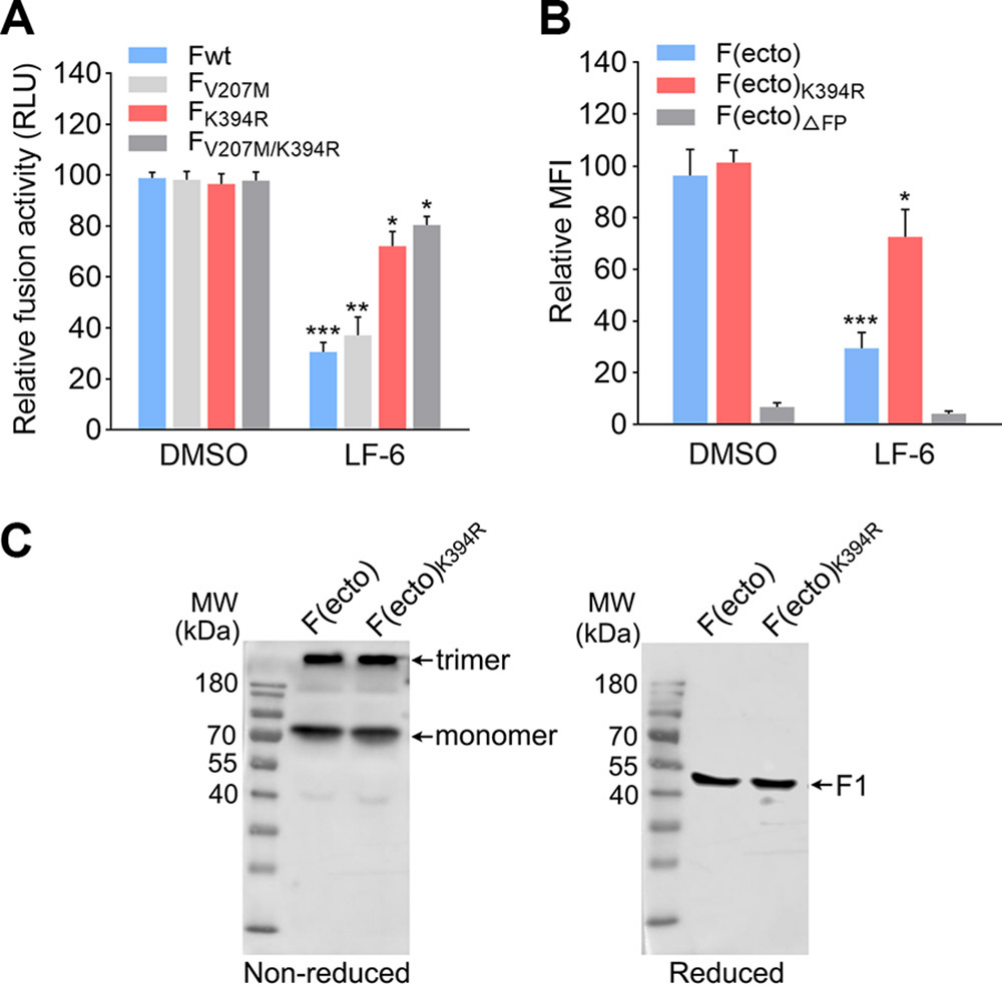
Investigation of the specific target and underlying mechanism of LF-6 against RSV. (A) Inhibitory effect of LF-6 on cell-cell fusion mediated by wild-type F or F variants. A dual-luciferase reporter assay was conducted to detect the membrane fusion activity in HEK293T cells that were transfected with plasmids expressing F_wt_, F_V207M_, F_K394R_, and F_V207M/K394R_, respectively. Data are mean ± SD; *n *=* *3 biological replicates. *, *P < *0.05; **, *P < *0.01; ***, *P < *0.001. (B) Inhibitory effect of LF-6 on the association between F(ecto), F(ecto)_K394R_, or F(ecto)_ΔFP_ and the cell membrane. HEp-2 cells were incubated with the F variants for 1 h at 37°C in the presence of LF-6 (20 μM) or DMSO, followed by staining with anti-RSV monoclonal antibody and Alexa Fluor 488-conjugated secondary antibody, respectively. MFI associated with the amount of F protein on the cell membrane was measured by flow cytometry. Data are mean ± SD; *n *=* *3 biological replicates. *, *P < *0.05; ***, *P < *0.001. (C) Supernatants of HEK293T cells transiently expressing F or F variants were separated by nonreducing or reducing SDS-PAGE, followed by Western blotting with motavizumab staining.

Next, the dual-luciferase reporter assay was conducted to evaluate the inhibitory effect of LF-6 on cell-cell fusion mediated by wild-type F (F_wt_) or F variants (e.g., F_V207M_, F_K394R_, and F_V207M/K394R_). We observed that LF-6 significantly reduced cell-cell fusion activity of F_wt_-transfected cells, whereas it has only a minor effect on F_K394R_- or F_V207M/K394R_-transfected cells (Fig. 4A). Moreover, there was no significant difference in cell-cell fusion activity between F_K394R_- and F_V207M/K394R_-transfected cells in the presence of LF-6, suggesting that K394R mutation was primarily responsible for the increased resistance of the mutant viruses (RSV F_K394R_ and RSV F_V207M/K394R_) against LF-6.

Subsequently, the effect of LF-6 on the association of soluble F proteins with the cell membrane was assessed by neutralization assay. In this assay, the ectodomain of F protein [i.e., F(ecto), F(ecto)_K394R_, and F(ecto)_ΔFP_] were expressed with a polyhistidine tag at the C terminus and purified in a high-ionic-strength buffer (29). As previously reported, soluble F(ecto) in a high-molarity buffer could maintain a pretriggered state and associate with the cell membrane by inserting fusion peptide into lipid bilayers of the host cell (16, 29, 31). In our study, we observed that both soluble F(ecto) and F(ecto)_K394R_ adopt trimeric or monomeric conformation in a nonreducing buffer (Fig. 4B and C). In contrast, only F1 fragments of F(ecto) or F(ecto)_K394R_ in reducing conditions were detected by immunoblotting. As expected, both F(ecto) and F(ecto)_K394R_ bound to the cell membrane, but F(ecto)_ΔFP_ lacking fusion peptide failed to associate with the cell membrane (Fig. 4B). In the presence of LF-6, the amount of F(ecto) on the cell membrane was substantially decreased, whereas the amount of F(ecto)_K394R_ on the cell membrane was only slightly decreased.

**FIG 5:**
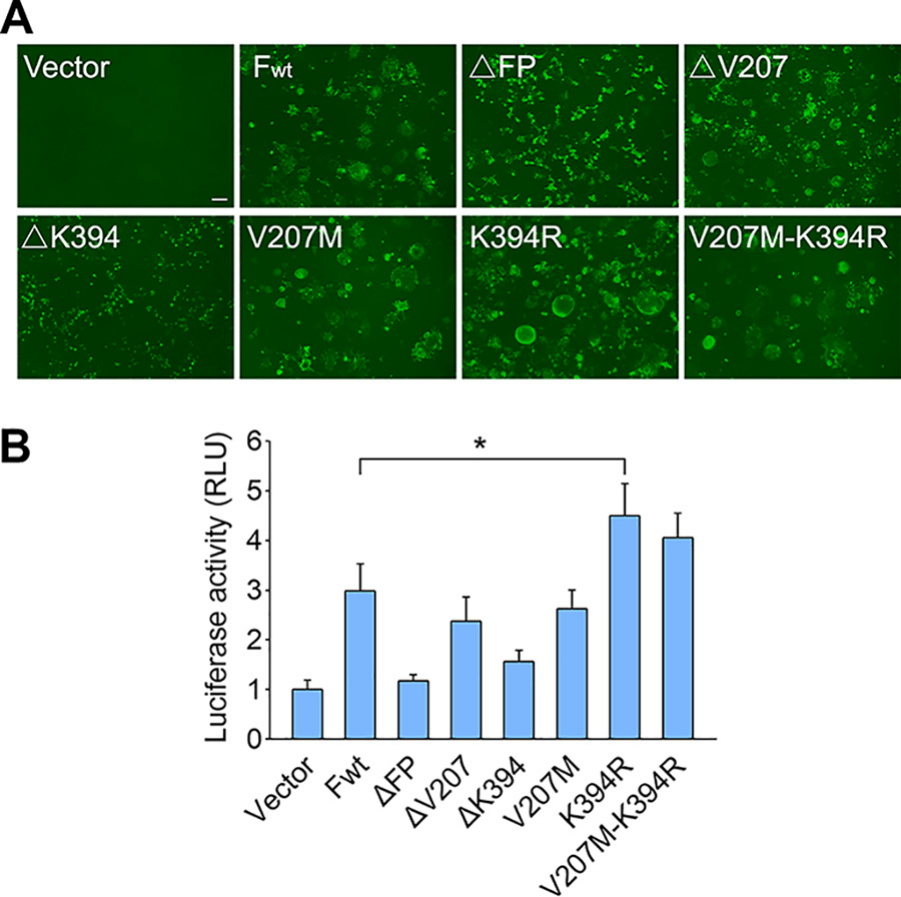
Deletion of K394 in RSV F protein results in its loss of fusion activity for syncytium formation. (A) HEK293T cells were transfected with plasmids expressing F_wt_ or F variants with ΔFP, ΔV207, ΔK394, V207M, K394R, or V207M/K394R mutations. At 48 h posttransfection, the F_wt_ or F variants on the cell surface were stained by anti-RSV F monoclonal antibody and Alexa Fluor 488-conjugated secondary antibody and then photographed under a fluorescence microscope. Bar, 100 μm. (B) Cell-cell fusion activity in transfected cells was determined by dual-luciferase reporter assay. Data are mean ± SD; *n *=* *3 biological replicates. *, *P < *0.05.

Finally, HEK293T cells were transfected with wild-type F or F variants. At 48 h posttransfection, the syncytia in transfected cells were stained with the anti-RSV monoclonal antibody, and the fusion activity in these cells was determined by dual-luciferase reporter assay. We found that fusion activity and syncytia numbers in K394R-transfected cells were increased compared to those in F_wt_-transfected cells (Fig. 5A and B), indicating that the K394R mutation enhanced the membrane fusion activity of F protein, whereas the V207M mutation in F protein did not contribute to membrane fusion in addition to syncytium formation. We next observed that deletion of fusion peptide (ΔFP) or residue K394 resulted in the loss of membrane fusion ability of the F protein, evidenced by low fusion activity and hardly any syncytia in the cells transfected with ΔFP or ΔK394 variants, suggesting that both fusion peptide and the K394 residue are indispensable for F protein to mediate membrane fusion. In contrast, the F variant with a deletion of V207 still produced large numbers of syncytia in cells, suggesting that V207 is not required for membrane fusion.

Taken together, the results described above suggest that (i) LF-6 inhibits RSV entry and infection by blocking the virus-cell and cell-cell fusion mediated by the viral F protein, (ii) the K394R mutation in RSV F protein is primarily responsible for viral resistance to LF-6, and (iii) the K394 residue is essential for RSV F protein to mediate membrane fusion.

### Substitutions at 394th residue alter the cross-resistance of RSV F protein against fusion inhibitors, as well as its membrane fusion activity.

To determine whether K394R in F protein confers cross-resistance to structurally distinct RSV fusion inhibitors, cells were infected with K394R mutant virus or wild-type virus in the presence of inhibitors. As shown in Table 1, the viral variant with K394R mutation in F protein showed cross-resistance to all the tested inhibitors except for ribavirin, a nucleoside analogue that inhibits viral RNA or DNA replication. In particular, the K394R variant conferred 6,024-fold resistance to JNJ-53718678, a potent RSV fusion inhibitor currently in phase 2 clinical trial (ClinicalTrials registration no. NCT04056611), compared to that of the wild-type virus. This inhibitor was synthesized by structure optimization from BMS-433771. Despite K394R not being included in the escape mutations for JNJ-53718678 that were recently reported, the K394R mutant virus exhibited more resistance to JNJ-53718678 than to BMS-433771, which elicited K394R mutation in its escape variants (32). AK-0529, a small-molecule fusion inhibitor of RSV also undergoing clinical trials, exhibited lower efficacy against K394R mutant virus than against wild-type virus. These results suggest that K394R represents a broad-spectrum resistance mutation for RSV fusion inhibitors.

**TABLE 1:** Antiviral activities of RSV F inhibitors against wild-type RSV or RSV variants harboring K394R mutation

Inhibitor	EC_50_^*a*^ (nM)		Fold resistance^*b*^	Reported escape mutations in F protein
	RSV-F_wt_	RSV-F_K394R_		
JNJ-53718678	0.83	5,000	6,024	L141W, D489Y
BMS-433771	10.51	20,000	1,902	F140I, V144A, D392G, K394R, D489Y
AK-0529	4.22	1,500	355	D486N, D489V, D489Y
TMC-353121	2.42	2,500	1,033	K394R, S398L, D486N
GS-5806	0.36	1.6	4	L138F, F140L, F488L, F488S, N517I
LF-6	2,780	>200,000	>71	V207M, K394R
Ribavirin	12,000	12,500	1	NA^*c*^

a EC_50_ is the concentration of inhibitor that reduced 50% of the cytopathic effect (CPE).

b Fold resistance is the fold change of EC_50_.

c NA, not applicable.

**FIG 6:**
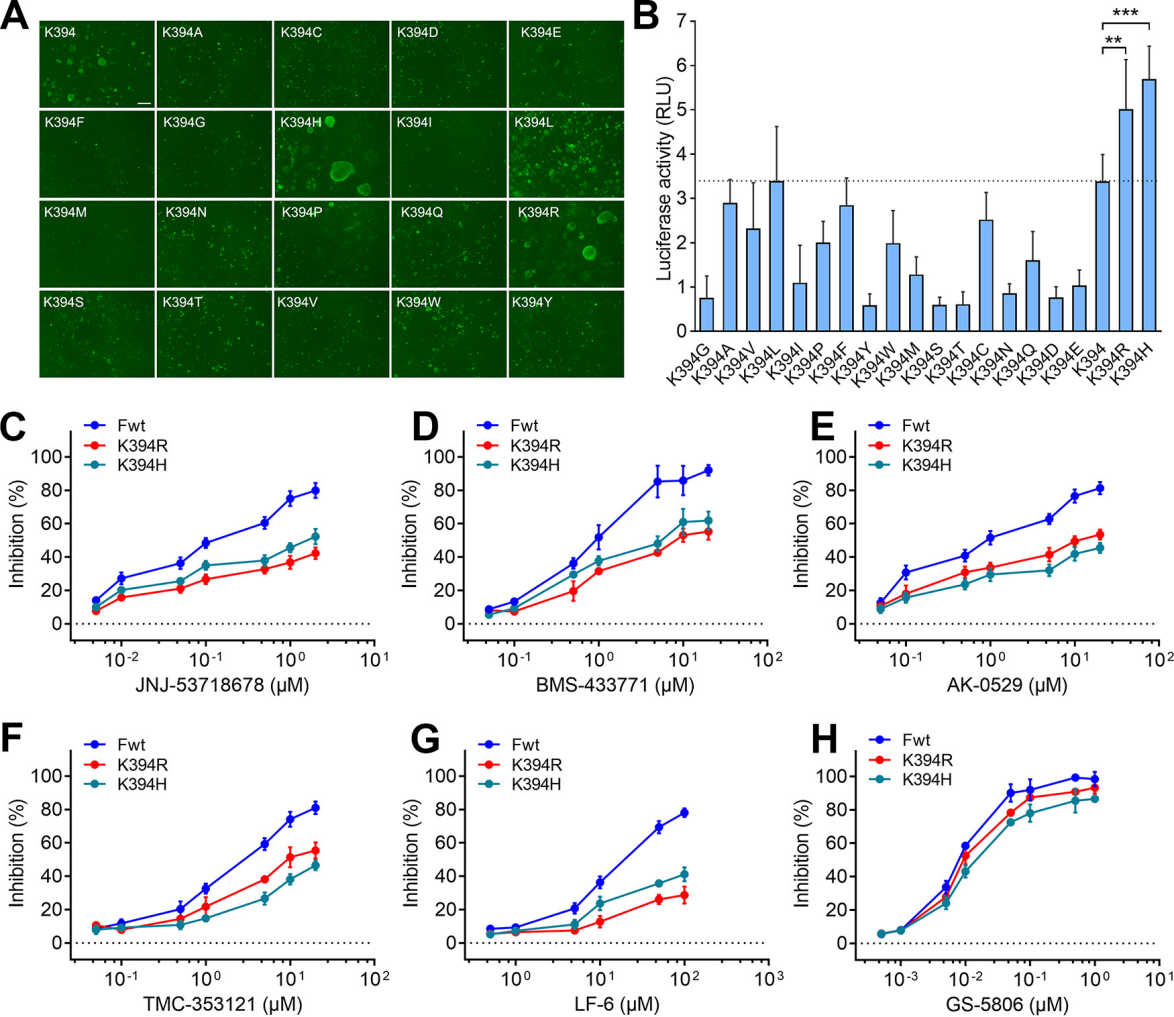
Substitutions at 394th residue of the RSV F protein alter its cell-cell fusion activity and resistance against fusion inhibitors. (A) HEK293T cells were transfected with plasmids encoding F_wt_ or F variants containing various amino acid substitutions at the 394th site in the F protein. At 48 h after transfection, the cells were incubated with anti-RSV monoclonal antibody and Alexa Fluor 488-conjugated secondary antibody, respectively. The cell-cell syncytia were observed under a fluorescence microscope. Bar, 100 μm. (B) Quantitative cell-cell fusion activity in the cells transfected with F_wt_ or F variants, determined by dual-luciferase reporter assay. The activities of firefly luciferase and *Renilla* luciferase were measured after transfection of plasmids expressing F_wt_ or F variants. Data are mean ± SD; *n *=* *3 biological replicates. **, *P < *0.01; ***, *P < *0.001. (C to H) HEK293T cells were transfected with plasmids expressing F_K394R_ or F_K394H_ in the presence of RSV fusion inhibitors. The cell-cell fusion activity was determined at 72 h after transfection. Data are mean ± SD; *n *=* *3 biological replicates.

To investigate whether amino acid substitutions at the 394th residue could alter the resistance of F protein against RSV fusion inhibitors, the K394 residue was substituted by 19 common amino acids. We observed that most of the substitutions in RSV F resulted in its loss of membrane fusion ability (Fig. 6A). Substantial syncytia were only generated in the cells that were transfected with F variants with K394 (wt), K394H, or K394R mutation. Although cell-cell fusion was activated in the cells that were transfected with K394L, no distinguishable syncytium was observed in these cells. Furthermore, the luciferase activities in K394H- or K394R-transfected cells were significantly higher than that in K394-transfected cells, as well as higher than those in cells transfected with other 17 substitution variants (Fig. 6B). Remarkably, the lysine (K), histidine (H), and arginine (R) are the only 3 positively charged (polar basic) amino acids among 20 common amino acids, suggesting that a positive charge at the 394th site is important for RSV F protein to mediate membrane fusion. Presumably, the microdomain around the 394th residue represents an active pocket for F protein inhibitors. Both K394R and K394H mutations in F protein exhibited considerable resistance to JNJ-53718678, BMS-433771, AK-0529, TMC-353121, and LF-6, and show only slight resistance to GS-5806 (Fig. 6C to F). The increased resistance caused by K394R was approximately equivalent to that caused by K394H. These results were consistent with the fact that K394R induced substantial viral resistance against BMS-433771 and TMC-353121 but only conferred 4.4-fold resistance to GS-5806 (Table 1), whose escape variants did not include the K394R mutation in previous reports.

### Mechanism of cross-resistance to fusion inhibitors conferred by the K394R mutation.

**FIG 7:**
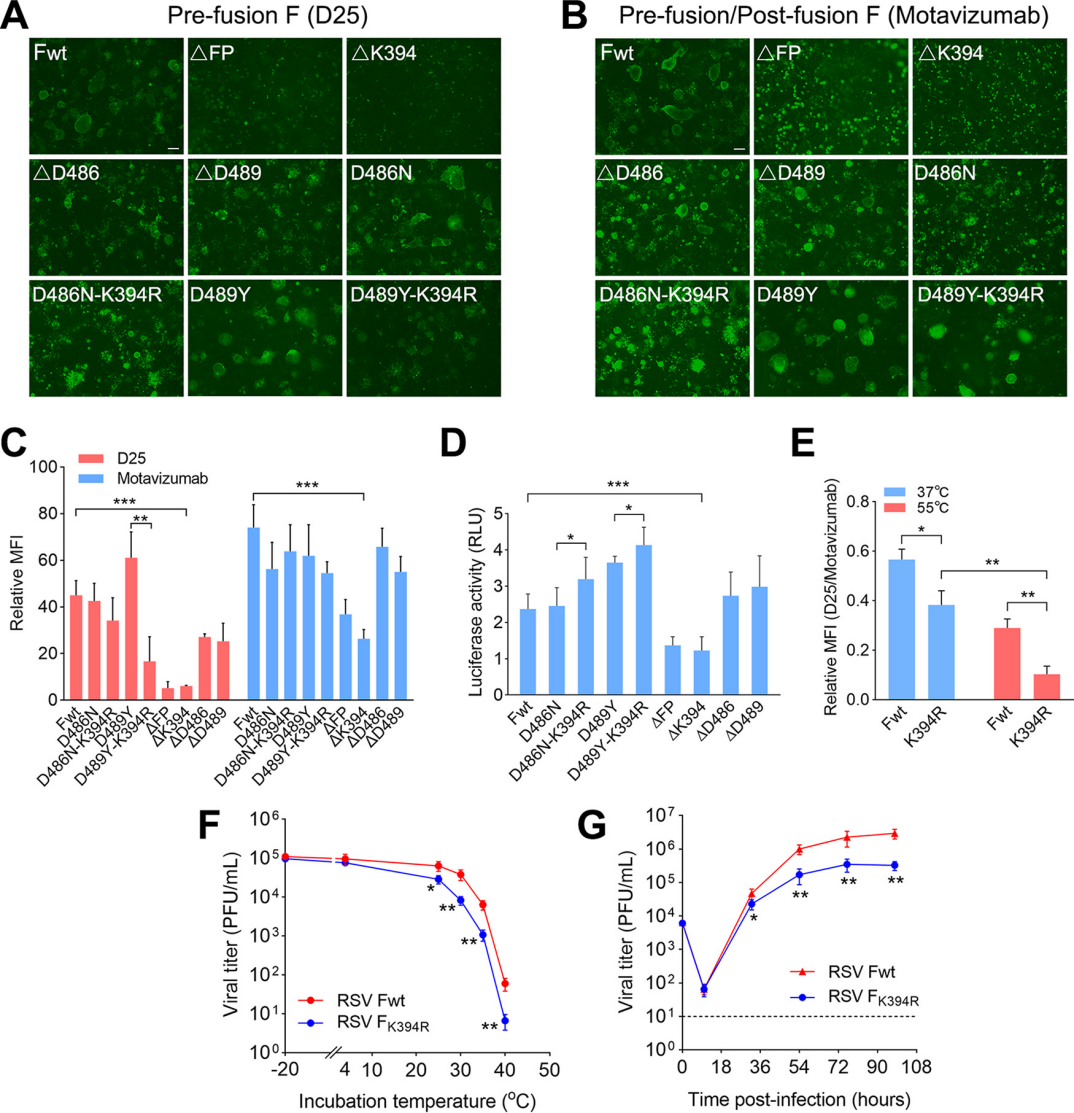
Effects of K394R mutation on the membrane fusion activity and stability of F protein. (A, B) Escape mutations altered the proportion of prefusion F to total F protein (prefusion F and postfusion F) on the cell surface. HEK293T cells were transfected with plasmids carrying F_wt_ or F variants. At 48 h posttransfection, the cells were stained with D25 or motavizumab monoclonal antibody, followed by incubation with Alexa Fluor 488-conjugated secondary antibody. Bar, 100 μm. (C) MFI of the transfected cells was analyzed using ImageJ software. Data are mean ± SD; *n *=* *3 biological replicates. **, *P < *0.01; ***, *P < *0.001. (D) Membrane fusion activity of transfected cells was determined by dual-luciferase reporter assay. Data are mean ± SD; *n *=* *3 biological replicates. *, *P < *0.05; ***, *P < *0.001. (E) Proportion of prefusion F to total F protein on the cell surface at 37°C or 55°C. HEK293T cells were transiently transfected with plasmids encoding F or F variants. At 48 h later, the cells were heat-shocked at 55°C for 10 min, followed by staining with D25 or motavizumab, and then incubated with Alexa Fluor 488-conjugated secondary antibody. MFI of the transfected cells was calculated. Data are mean ± SD; *n *=* *3 biological replicates. *, *P < *0.05; **, *P < *0.01. (F) Thermal stability of the wild-type virus and the K394R mutant. RSV virions were incubated at different temperatures for 24 h in the absence of cells. The remaining viral titers were determined by plaque assay. Data are mean ± SD; *n *=* *3 or 4 biological replicates. *, *P < *0.05; **, *P < *0.01. (G) Growth curves of wild-type RSV or the RSV variant with a K394R mutation. HEp-2 cells were infected with RSV or RSV-F_K394R_. At indicated hours after the viral infection, virus suspensions were harvested and subjected to viral titer determination by plaque assay. Data are mean ± SD; *n *=* *3 or 4 biological replicates. *, *P < *0.05; **, *P < *0.01.

The K394R is an escape mutation for RSV fusion inhibitors. However, the molecular mechanism for K394R to mediate cross-resistance against these inhibitors is currently unknown. In the next series of experiments, the effects of K394R mutation on the conformation stability and the membrane fusion activity of RSV F protein were determined using D25 and motavizumab, which are prefusion F- and prefusion F/postfusion F-specific antibodies, respectively. We found that deletion of K394 (ΔK394) or fusion peptide (ΔFP) significantly reduced the proportion of prefusion F to total F protein in the cells (Fig. 7A to C). Moreover, no syncytia were observed in these cells, whereas substantial syncytia existed in the cells transfected with ΔD486 or ΔD489 variants. These results suggest that K394 is crucial for F protein to mediate membrane fusion, as well as for conformational stability of prefusion F. In contrast, D486 and D489 residues are not required for F protein to mediate membrane fusion (Fig. 7D). The proportion of prefusion F in the cells transfected with D486N/K394R and D489Y/K394R variants was lower than that in cells transfected with D486N and D489Y variants, respectively (Fig. 7C), suggesting that the K394R mutation reduced the stability of prefusion F. Furthermore, we also observed that an additional K394R mutation in D486N and D489Y variants could enhance the membrane fusion activity of F protein (Fig. 7D). When heat-shocked at 55°C, K394R increased the shift of prefusion F into postfusion F (Fig. 7E). These results suggest that K394R mutation reduced the stability of F protein and increased the triggering rate of F protein, resulting in enhanced membrane fusion activity.

To investigate whether K394R mutation in F protein increases the sensitivity of RSV to thermal inactivation, we incubated the mutant virus or wild-type virus at different temperatures for 24 h in the absence of cells. Both the K394R mutant virus and the wild-type virus showed no obvious changes in viral titers after incubation at 4°C. However, the titer of the K394R mutant virus was rapidly reduced when the viral variant was incubated at a temperature over 25°C, and decreased more quickly than the titer of the wild-type virus (Fig. 7F). After incubation at 40°C for 24 h, the titer of the K394R mutant virus was decreased over 14,000-fold compared to the titer of the mutant virus that was incubated at −20°C. In contrast, only an 1,800-fold reduction of viral titer was observed for wild-type virus after incubation at 40°C. These results suggest that K394R in the F protein decreased the thermal stability of the mutant virions.

To determine the effect of the K394R mutation on viral fitness, viral titers in RSV-infected cells were assessed at indicated intervals after infection. The viral variants harboring the K394R mutation did not grow as well as the wild-type virus *in vitro* and was nearly 10-fold less infectious than wild-type virus after infection for 98 h (Fig. 7G). These results indicate that the K394R mutation leads to a reduction in viral fitness.

**FIG 8:**
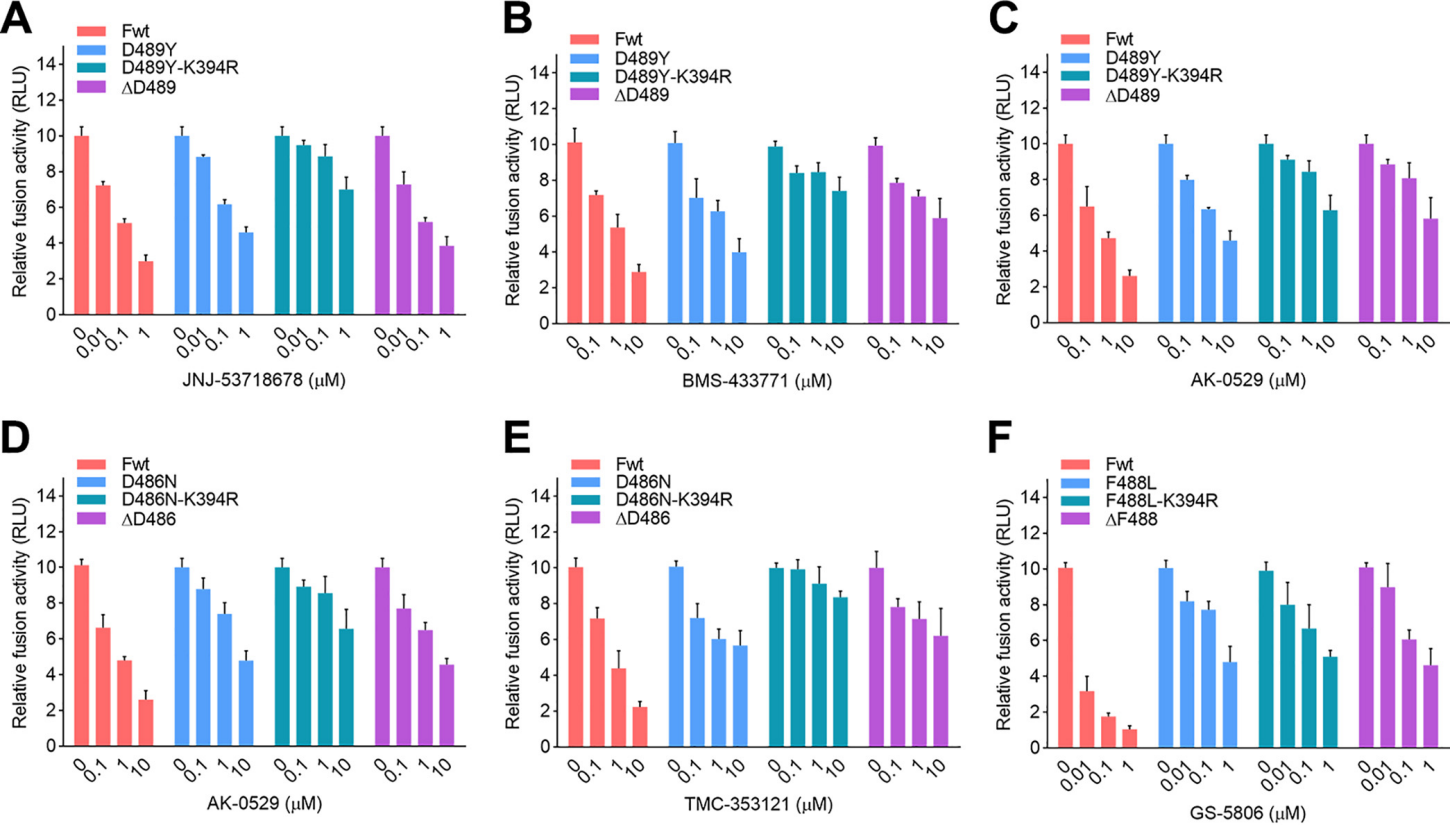
Cooperative effects of escape mutations for RSV F inhibitors on resistance. HEK293T cells were transfected with plasmids harboring escape mutations for RSV F inhibitors. At 24 h posttransfection, the cells were treated with JNJ-53718678 (A), BMS-433771 (B), AK-0529 (C, D), TMC-353121 (E), and GS-5806 (F), respectively. Cell-cell fusion activity was determined at 72 h posttransfection. Data are mean ± SD; *n *=* *3 biological replicates.

Additionally, the K394R mutation showed cooperative effects with other escape mutations, including D486N or D489Y, in increasing the resistance of F protein against RSV fusion inhibitors (Fig. 8A to E). The D486N or D489Y single mutation in the F protein resulted in moderate resistance against these inhibitors. Notably, an additional K394R mutation in the D486N or D489Y variants dramatically increased resistance of the F variants, which exhibited stronger resistance than the ΔD486 and ΔD489 variants against the tested inhibitors.

## DISCUSSION

The F protein is essential for RSV infectivity and can serve as a valuable target for anti-RSV therapy. Some small molecules targeting the RSV F protein, such as BMS-433771, TMC-353121, JNJ-2408068, JNJ-53718678, GS-5806, and AK-0529, have shown promising prospects in anti-RSV drug development (17–24). These inhibitors exhibit potent *in vitro* and *in vivo* anti-RSV activity. Several of them (e.g., JNJ-53718678, GS-5806, and AK-0529) are undergoing clinical development. However, almost all of the small-molecule RSV F inhibitors that have been reported are associated with the risk of inducing viral resistance, which may lead to loss of their therapeutic benefits in the clinic. Therefore, understanding their resistance mutations and resistance mechanism will facilitate recognizing possible resistance risks before their clinical application.

This study demonstrated a dual-luciferase protocol for viral fusion inhibitor discovery and identified LF-6 as a new small-molecule RSV fusion inhibitor using this protocol. LF-6 suppressed RSV infection by blocking virus-cell and cell-cell fusion, and its target has been proved to be the viral F protein through a series of experiments *in vitro*. Sequence analysis of its escape variants indicated that K394R mutation, an escape mutation for several reported RSV inhibitors (e.g., BMS-433771 and TMC-353121), also occurred in the viral variants conferring resistance to LF-6. Despite the fact that K394R emerged as a common mutation for small-molecule inhibitors with different structures (18, 26), its effects of inducing viral resistance have been largely neglected mainly due to its location at domain I/II, a subunit with unclear effects for viral fusion. Moreover, whether K394R mediates broad-spectrum resistance against RSV fusion inhibitors, especially against the inhibitors that are currently in clinical trials, has not yet been reported. Our results demonstrated that viral variants harboring a single K394R mutation in the F protein showed cross-resistance to several promising candidates (BMS-433771, TMC-353121, JNJ-53718678, and AK-0529), as well as against the inhibitor identified in this study, LF-6. Notably, the K394R mutant virus conferred 6,024-fold and 355-fold resistance to JNJ-53718678 and AK-0529, respectively, both of which are currently in phase 2 clinical trials. Thus far, the resistant isolates of these two inhibitors did not generate a K394R mutation in previous reports, but we should raise concerns for the possible risk in clinical therapy of emerging resistant virus harboring K394R mutation.

Escape variants for each inhibitor generally harbor several mutations in the viral F protein. The viral resistance caused by these mutations is different. It is important to understand which mutations are primarily responsible for viral resistance or whether these mutations have cooperative effects. Previous studies demonstrated that D486N and D489Y are major escape mutations for RSV F inhibitors (e.g., TMC-353121, BMS-433771, and AK-0529) through a direct escape mechanism (22). Remarkably, our results indicated that deletion of D486 and D489 did not reduce the fusion activity of the F protein. The F variants lacking D486 or D489 only exhibited slight resistance to these inhibitors. In contrast, deletion of K394 in the F protein resulted in its loss of fusion activity. Additional K394R mutation in D486N- or D489Y-mutated F variants dramatically reduced the inhibitory effects of these inhibitors on the membrane fusion process mediated by the F variants. Furthermore, the viral variant carrying a K394R mutation exhibited low susceptibility to these inhibitors. These findings suggest that the K394R mutation is presumably a major determinant for viral cross-resistance. The combination of K394R with other escape mutations leads to cooperative effects on resistance.

In addition to the K394R mutation, substitutions of K394 (lysine) into H (histidine) in F protein were also associated with cross-resistance to RSV fusion inhibitors (e.g., BMS-433771, TMC-353121, JNJ-53718678, and AK-0529). However, other residue substitutions, except for 3 positively charged residues (K, H, and R) at the 394th site, substantially attenuated the fusion activity of F protein and did not show resistance to these inhibitors, suggesting that a positively charged residue at the 394th site is important for the F protein to mediate membrane fusion as well as for viral cross-resistance. Neutralizing the positive charge at residue 394 in the F protein may represent a potential strategy to design more potent inhibitors with lower resistance risk. Further investigations are needed to clarify the molecular mechanism for 394th-position positively charged residues in altering membrane fusion activity of F protein.

We noted that deletion of fusion peptide or K394 in the F protein resulted in substantial postfusion F on the cell surface, whereas hardly any prefusion F protein and no syncytia occurred in these cells. However, deletion of D486 or D489 showed little effect on the fusion activity of the F protein, and only slightly decreased the proportion of prefusion F on the cell surface compared to wild-type F. Clearly, K394 residue and fusion peptide are indispensable for the F protein to mediate membrane fusion. Both of them are important for the formation of prefusion F in cells. In contrast, D486 or D489 residues are not required for membrane fusion. Future design of RSV F inhibitors to target the residues that are essential for the fusion ability of F protein is suggested.

Our results demonstrated that a single K394R mutation or an additional K394R mutation in D486N- or D489Y-mutated F variants increased the triggering rate of F protein, thus enhancing the membrane fusion activity of the F protein, as well as decreased the proportion of prefusion F on the cell surface. The instability of the F protein resulted in a shorter time window for inhibitors to bind and increased viral resistance against these inhibitors. It is reasonable to conclude that the K394R mutation mediates cross-resistance by an indirect mechanism of kinetic escape. Further studies are needed to investigate whether K394R mutation confers broad-spectrum resistance to RSV F inhibitors *in vivo*, as well as to develop effective strategies or alternative inhibitors to overcome the resistance problems.

## MATERIALS AND METHODS

### Cells, viruses, and compounds.

HEp-2 and HEK293T cells were purchased from the American Type Culture Collection (ATCC) and maintained in Dulbecco’s modified Eagle’s medium (DMEM) supplemented with 10% fetal bovine serum (FBS) and 2 mM l-glutamine. RSV A2 was obtained from Wuhan University, China. RSV A2-F_K394R_ was isolated and identified in our previous study (29). All the viruses were grown in HEp-2 cells and stored at −70°C. Viral titers were determined by plaque assay. Salvianolic acid R (LF-6) was isolated from the aerial part of *Mesona chinensis* Benth. (Lamiaceae) by the authors, following our previously reported procedure (30). The purity of LF-6 (>98%) was validated through high-performance liquid chromatography (HPLC) analysis. Ribavirin, heparin, and BMS-433771 were purchased from Sigma-Aldrich, Inc. TMC-353121, JNJ-53718678, GS-5806, and AK-0529 were purchased from MedChem Express, Inc.

### Plasmids.

Human codon-optimized full-length viral wild-type F (F_wt_), F_V207M_, F_K394R_, F_D486N_, F_D489Y_, F_K394R/D486N_, F_K394R/D489Y,_ F_ΔV207_ (deletion of V207 in F_wt_), F_ΔK394_ (deletion of K394 in F_wt_), F_ΔD486_ (deletion of D486 in F_wt_), F_ΔD489_ (deletion of D489 in F_wt_), F_ΔFP_ (deletion of residues FLGFLLGVGS in fusion peptide), and residue-substituted F (various residues substituted at the 394th residue of F_wt_) were each respectively cloned into pcDNA3.1(+) vector. The pT7-Luc plasmid carries a firefly luciferase gene under the control of the T7 promoter. The pCAG-T7 Pol plasmid can express T7 RNA polymerase, which specifically binds to the T7 promoter of pT7-Luc and initiates expression of firefly luciferase. pRL-TK carries a *Renilla* luciferase gene.

### Cell-cell fusion assay.

HEK293T cells were dissociated from cell culture flasks and divided into two groups. One group of cells were transiently cotransfected with pT7-Luc plasmids and pcDNA3.1(+) encoding RSV F or RSV F variants. Another group of cells was cotransfected with pRL-TK and pCAG-T7 Pol plasmids. All of the plasmids were transiently transfected into HEK293T cells using Lipofectamine 2000 reagent (Thermo Fisher Scientific, CA, USA) in Opti-MEM. At 5 h after transfection, the Opti-MEM on the cells was discarded and supplemented with fresh cell culture medium. After incubation for 24 h, the two groups of cells were mixed in equal proportion and seeded into 96-well plates in the presence of inhibitors at various concentrations. At 72 h posttransfection, the activities of firefly luciferase and *Renilla* luciferase were measured using the dual-luciferase reporter assay system (Promega, WI, USA).

### Cell viability assay.

HEp-2 cells were grown in 96-well plates and incubated overnight at 37°C. The cell supernatants were discarded and supplemented with culture medium containing different concentrations of compounds. After 72 h, cell supernatants in each well were removed and supplemented with 100 μl DMEM containing 10 μl of Cell Counting Kit-8 (Sigma-Aldrich). After 2 h at 37°C, cell viability was calculated according to the absorbance at 450 nm using a Multiskan Spectrum instrument.

### Plaque assay.

HEp-2 cells were infected with 100 50% tissue culture infective dose (TCID_50_) of RSV A2 in the presence of various concentrations of compounds. At 48 h postinfection, cell supernatants were harvested and diluted to a range of concentrations from 10^−1^ to 10^−6^. The virus dilutions were added to a new 24-well plate of HEp-2 cells for viral titer determination. At 2 h after infection, cells were washed twice with phosphate-buffered saline (PBS) to remove unbound virions on the cell surface, followed by coverage of 500 μl 1.5% agarose in maintenance medium (2% FBS). After agarose solidification, 500 μl of maintenance medium was added to each well. On day 4 or 5 postinfection, cells were fixed with 4% formaldehyde in PBS and then stained with 1% crystal violet for 1 h at room temperature (RT). After washing with PBS, the virus-induced plaques were counted.

### Immunofluorescence assay.

HEp-2 cells or HEK293T cells were seeded into 24-well plates and incubated overnight. For viral infection assay, HEp-2 cells were infected with RSV A2 at a multiplicity of infection (MOI) of 0.5 in the presence of LF-6 or ribavirin. At 48 h postinfection, cells were fixed with 4% paraformaldehyde in PBS and permeabilized with 0.1% Triton X-100 for 10 min at RT. Cells were then incubated with RSV F-specific antibody (catalog no. ab94968; Abcam, USA) in PBS containing 4% bovine serum albumin (BSA) at RT, followed by staining with DyLight 594-conjugated secondary antibody (Thermo Fisher Scientific) for 2 h. After washing twice with PBS, cells were stained with 4′,6-diamidino-2-phenylindole (DAPI) for 10 min at RT. Cells were washed three times with PBS and then photographed using a fluorescence microscope. For transfection assay, HEK293T cells were transfected with plasmids encoding various F variants by Lipofectamine 2000 reagent at 24 h after transfection. At 48 h posttransfection, the cells were incubated with RSV F-specific antibody (catalog no. ab94968; Abcam), D25 (catalog no. PABL-322; Creative Biolabs), or Motavizumab (catalog no. TAB-709; Creative Biolabs) for 2 h at RT, and then stained with Alexa 488-conjugated secondary antibody (catalog no. A11013; Thermo Fisher Scientific) for 1 h at RT. After washing three times with PBS, cells were photographed under a fluorescence microscope.

### Time-of-addition assay.

HEp-2 cells were grown in 96-well plates and incubated overnight. The cells were infected with RSV and treated with LF-6 (10 μM) or ribavirin (10 μM) at the indicated time points after exposure of cells to the virus. At 40 h postinfection, viral stocks were prepared from cells and subjected to viral titer determination by plaque assay.

### Attachment assay.

HEp-2 cells grown in flasks were dissociated and then centrifuged for 5 min at 4°C and 1,500 × *g*. The pelleted cells were resuspended in 4°C precooled medium containing the mixtures of virus suspension with LF-6 or heparin at different concentrations. Afterward, the cells were gently rocked for 1 h at 4°C to allow viral attachment to the cell membrane. Following the incubation, the unbound virus on the cell surface was removed by centrifugation at 2,000 × *g* at 4°C for 3 min and then washed twice with PBS. Afterward, cells were fixed with 4% paraformaldehyde for 20 min at 4°C. Following the washing twice with PBS, cells were incubated with mouse anti-RSV F monoclonal antibody (catalog no. ab94968; Abcam) for 2 h at RT and followed by staining with Alexa Fluor 488-conjugated anti-mouse antibody (Thermo Fisher Scientific) at RT for 1 h. Finally, cells were washed twice with PBS and subjected to flow cytometry (BD Biosciences, CA, USA). Data were analyzed using FlowJo v.7.6.

### Virus-cell fusion assay.

HEp-2 cells in cell culture flasks were infected with RSV A2. When 80% to 90% of cells reached apoptosis, the cells were collected, freeze-thawed, and centrifuged at 4°C and 2,000 × *g* for 20 min. The virus suspension was collected in a 50-ml conical tube containing 10 ml glycerol mixtures (30% glycerol and 50 mM HEPES [pH 7.4]), then centrifuged at 4°C and 24,000 × *g* for 3 h with an SW28 rotor. The pelleted virus was resuspended in 600 μl of Opti-MEM. For the confocal assay, octadecylrhodamine B chloride (R18; Thermo Fisher Scientific) and 3,3′-dioctadecyloxacarbocyanine perchlorate (DiOC_18_, Thermo Fisher Scientific) were diluted to 500 μM and then mixed at a proportion of 1:1. The purified virus in 2.5 ml Opti-MEM (100 μg/ml) was incubated with 15 μl of the probe mixtures. After incubation at 4°C for 1 h, the labeled virus was passed through a 0.25-μm filter syringe filter (Millipore, MA, USA). HEp-2 cells were infected with the labeled virus (MOI = 3) and treated with LF-6 (10 μM or 20 μM), heparin (4 μM), or ribavirin (20 μM) at 0, 20, 40, and 60 min postinfection. Two hours later, cells were fixed with 4% paraformaldehyde for 25 min and washed twice with PBS, followed by staining with DAPI for 5 min. After washing twice with PBS, the fluorescence emitted by the probes was observed using a confocal microscope. For the flow cytometry assay, the virus was incubated with R18 probes at 2 μM for 30 min at RT and passed through a 0.25-μm filter syringe filter. HEp-2 cells were dissociated from flasks and pretreated with LF-6, ribavirin, or heparin before R18-RSV infection. At 2 h after viral infection, cells were fixed with 4% paraformaldehyde for 20 min at RT. The fluorescence intensity emitted by R18 probes was detected by flow cytometry.

### Western blot assay.

The ectodomains of RSV A2 F [i.e., F(ecto)] and F variants [i.e., F(ecto)_ΔFP_ and F(ecto)_K394R_] were codon-optimized for expression in mammalian cells and cloned into pcDNA3.1(+) vector. F(ecto) contains amino acid (aa) residues 1 to 526 with a 6×His tag at the C terminus, whereas F(ecto)_ΔFP_ and F(ecto)_K394R_ were derived from F(ecto) with the deletion of residues 137 to 146 or the substitution of K394 to R394, respectively. All of the plasmids were transfected into HEK293T cells. On day 5 posttransfection, the soluble F proteins in cell culture supernatant were harvested and separated by nonreducing or reducing SDS-PAGE, then transferred to polyvinylidene fluoride (PVDF) membranes. The blotted proteins were stained with motavizumab, a monoclonal antibody that binds to both prefusion and postfusion forms of RSV F proteins, and then detected with a chemiluminescent agent using a chemiluminescence imager (Amersham Imager 600; GE Healthcare).

### Neutralizing assay.

This assay was performed similarly to one previously described (29). Briefly, LF-6 (20 μM) was premixed with soluble F(ecto), F(ecto)_K394R_, or F(ecto)_ΔFP_ at 4°C for 20 min. HEp-2 cells were incubated with the mixtures of LF-6 and soluble F proteins for 1 h at 37°C. Afterward, the cells were incubated with anti-RSV monoclonal antibody (catalog no. ab94968; Abcam) in PBS for 1 h, followed by staining with Alexa Fluor 488-conjugated anti-mouse antibody (Thermo Fisher Scientific) at 37°C for 1 h. Mean fluorescence intensity (MFI) correlated with the amount of F protein on the cell surface was measured by flow cytometry (BD Biosciences). All of the soluble proteins were used at a final concentration of 2 μg/ml. Data were analyzed using FlowJo v.10.

### Cell-surface triggering assay.

HEK293T cells were seeded in 6-well plates and incubated overnight at 37°C and 5% CO_2_. The cells were transiently transfected with plasmids encoding RSV F or RSV F variants using Lipofectamine 2000 reagent. At 48 h after transfection, cells were dissociated and heat-shocked at 55°C for 10 min and then incubated with anti-RSV F antibody (D25 or motavizumab) at 4°C overnight, followed by staining with Alexa Fluor 488-conjugated secondary antibody (Thermo Fisher Scientific) at RT for 2 h. Finally, cells were washed twice with PBS and subjected to flow cytometry (BD Biosciences). Mean fluorescence intensity (MFI) was calculated using FlowJo v.7.6.

### Viral temperature sensitivity and growth kinetics.

For the thermal stability assay, the K394R mutant virus and the wild-type virus were prepared into equal aliquots, which were frozen at −20°C or incubated at different temperatures for 24 h followed by a single freeze-thaw. The remaining infectivity was determined by plaque assay. For viral growth curves, HEp-2 cells were infected with the wild-type virus or the K394R variant. At indicated hours after infection, the virus suspensions in cells were harvested and subjected to viral titer determination by plaque assay.

### Statistical analysis.

Data in this study were presented as mean ± standard deviation (SD). Statistical analyses of the data were conducted with GraphPad Prism v.8.0. The two-tailed Student’s *t* test was used to measure the statistical difference between groups (GraphPad Software, Inc., San Diego, CA). *P* values of* ≤*0.05 were considered to represent a statistically significant difference between compared groups.

## References

[1] Shay DK, Holman RC, Roosevelt GE, Clarke MJ, Anderson LJ. 2001. Bronchiolitis-associated mortality and estimates of respiratory syncytial virus-associated deaths among US children, 1979–1997. J Infect Dis 183:16–22. 10.1086/317655. 11076709

[2] Falsey AR, Hennessey PA, Formica MA, Cox C, Walsh EE. 2005. Respiratory syncytial virus infection in elderly and high-risk adults. N Engl J Med 352:1749–1759. 10.1056/NEJMoa043951. 15858184

[3] Rainisch G, Adhikari B, Meltzer MI, Langley G. 2020. Estimating the impact of multiple immunization products on medically-attended respiratory syncytial virus (RSV) infections in infants. Vaccine 38:251–257. 10.1016/j.vaccine.2019.10.023. 31740097 PMC7029767

[4] Lambert L, Sagfors AM, Openshaw PJ, Culley FJ. 2014. Immunity to RSV in early-life. Front Immunol 5:466. 10.3389/fimmu.2014.00466. 25324843 PMC4179512

[5] Krilov LR. 2002. Safety issues related to the administration of ribavirin. Pediatr Infect Dis J 21:479–481. 10.1097/00006454-200205000-00037. 12150196

[6] Chu HY, Englund JA. 2013. Respiratory syncytial virus disease: prevention and treatment. Curr Top Microbiol Immunol 372:235–258. 10.1007/978-3-642-38919-1_12. 24362693

[7] Krusat T, Streckert HJ. 1997. Heparin-dependent attachment of respiratory syncytial virus (RSV) to host cells. Arch Virol 142:1247–1254. 10.1007/s007050050156. 9229012

[8] Kahn JS, Schnell MJ, Buonocore L, Rose JK. 1999. Recombinant vesicular stomatitis virus expressing respiratory syncytial virus (RSV) glycoproteins: RSV fusion protein can mediate infection and cell fusion. Virology 254:81–91. 10.1006/viro.1998.9535. 9927576

[9] Hall CB, Simoes EA, Anderson LJ. 2013. Clinical and epidemiologic features of respiratory syncytial virus. Curr Top Microbiol Immunol 372:39–57. 10.1007/978-3-642-38919-1_2. 24362683

[10] Techaarpornkul S, Barretto N, Peeples ME. 2001. Functional analysis of recombinant respiratory syncytial virus deletion mutants lacking the small hydrophobic and/or attachment glycoprotein gene. J Virol 75:6825–6834. 10.1128/JVI.75.15.6825-6834.2001. 11435561 PMC114409

[11] Karron RA, Buonagurio DA, Georgiu AF, Whitehead SS, Adamus JE, Clements-Mann ML, Harris DO, Randolph VB, Udem SA, Murphy BR, Sidhu MS. 1997. Respiratory syncytial virus (RSV) SH and G proteins are not essential for viral replication in vitro: clinical evaluation and molecular characterization of a cold-passaged, attenuated RSV subgroup B mutant. Proc Natl Acad Sci USA 94:13961–13966. 10.1073/pnas.94.25.13961. 9391135 PMC28415

[12] Schlender J, Zimmer G, Herrler G, Conzelmann KK. 2003. Respiratory syncytial virus (RSV) fusion protein subunit F2, not attachment protein G, determines the specificity of RSV infection. J Virol 77:4609–4616. 10.1128/jvi.77.8.4609-4616.2003. 12663767 PMC152164

[13] Calder LJ, Gonzalez-Reyes L, Garcia-Barreno B, Wharton SA, Skehel JJ, Wiley DC, Melero JA. 2000. Electron microscopy of the human respiratory syncytial virus fusion protein and complexes that it forms with monoclonal antibodies. Virology 271:122–131. 10.1006/viro.2000.0279. 10814577

[14] Sugrue RJ, Brown C, Brown G, Aitken J, Rixon HWM. 2001. Furin cleavage of the respiratory syncytial virus fusion protein is not a requirement for its transport to the surface of virus-infected cells. J Gen Virol 82:1375–1386. 10.1099/0022-1317-82-6-1375. 11369882

[15] McLellan JS, Chen M, Leung SM, Graepel KW, Du XL, Yang YP, Zhou TQ, Baxa U, Yasuda E, Beaumont T, Kumar A, Modjarrad K, Zheng ZZ, Zhao M, Xia NS, Kwong PD, Graham BS. 2013. Structure of RSV fusion glycoprotein trimer bound to a prefusion-specific neutralizing antibody. Science 340:1113–1117. 10.1126/science.1234914. 23618766 PMC4459498

[16] Chaiwatpongsakorn S, Epand RF, Collins PL, Epand RM, Peeples ME. 2011. Soluble respiratory syncytial virus fusion protein in the fully cleaved, pretriggered state is triggered by exposure to low-molarity buffer. J Virol 85:3968–3977. 10.1128/JVI.01813-10. 21307202 PMC3126149

[17] Cianci C, Langley DR, Dischino DD, Sun YX, Yu KL, Stanley A, Roach JL, Li ZF, Dalterio R, Colonno R, Meanwell NA, Krystal M. 2004. Targeting a binding pocket within the trimer-of-hairpins: small-molecule inhibition of viral fusion. Proc Natl Acad Sci USA 101:15046–15051. 10.1073/pnas.0406696101. 15469910 PMC523459

[18] Roymans D, De Bondt HL, Arnoult E, Geluykens P, Gevers T, Van Ginderen M, Verheyen N, Kim H, Willebrords R, Bonfanti JF, Bruinzeel W, Cummings MD, van Vlijmen H, Andries K. 2010. Binding of a potent small-molecule inhibitor of six-helix bundle formation requires interactions with both heptad-repeats of the RSV fusion protein. Proc Natl Acad Sci USA 107:308–313. 10.1073/pnas.0910108106. 19966279 PMC2806771

[19] Douglas JL, Panis ML, Ho E, Lin KY, Krawczyk SH, Grant DM, Cai R, Swaminathan S, Chen XW, Cihlar T. 2005. Small molecules VP-14637 and JNJ-2408068 inhibit respiratory syncytial virus fusion by similar mechanisms. Antimicrob Agents Chemother 49:2460–2466. 10.1128/AAC.49.6.2460-2466.2005. 15917547 PMC1140497

[20] Roymans D, Alnajjar SS, Battles MB, Sitthicharoenchai P, Furmanova-Hollenstein P, Rigaux P, Berg JVD, Kwanten L, Ginderen MV, Verheyen N, Vranckx L, Jaensch S, Arnoult E, Voorzaat R, Gallup JM, Larios-Mora A, Crabbe M, Huntjens D, Raboisson P, Langedijk JP, Ackermann MR, McLellan JS, Vendeville S, Koul A. 2017. Therapeutic efficacy of a respiratory syncytial virus fusion inhibitor. Nat Commun 8:167. 10.1038/s41467-017-00170-x. 28761099 PMC5537225

[21] Perron M, Stray K, Kinkade A, Theodore D, Lee G, Eisenberg E, Sangi M, Gilbert BE, Jordan R, Piedra PA, Toms GL, Mackman R, Cihlar T. 2015. GS-5806 inhibits a broad range of respiratory syncytial virus clinical isolates by blocking the virus-cell fusion process. Antimicrob Agents Chemother 60:1264–1273. 10.1128/AAC.01497-15. 26666922 PMC4776015

[22] Battles MB, Langedijk JP, Furmanova-Hollenstein P, Chaiwatpongsakorn S, Costello HM, Kwanten L, Vranckx L, Vink P, Jaensch S, Jonckers TH, Koul A, Arnoult E, Peeples ME, Roymans D, McLellan JS. 2016. Molecular mechanism of respiratory syncytial virus fusion inhibitors. Nat Chem Biol 12:87–93. 10.1038/nchembio.1982. 26641933 PMC4731865

[23] DeVincenzo JP, Whitley RJ, Mackman RL, Scaglioni-Weinlich C, Harrison L, Farrell E, McBride S, Lambkin-Williams R, Jordan R, Xin Y, Ramanathan S, O’Riordan T, Lewis SA, Li X, Toback SL, Lin S-L, Chien JW. 2014. Oral GS-5806 activity in a respiratory syncytial virus challenge study. N Engl J Med 371:711–722. 10.1056/NEJMoa1401184. 25140957

[24] McKimm-Breschkin JL, Jiang S, Hui DS, Beigel JH, Govorkova EA, Lee N. 2018. Prevention and treatment of respiratory viral infections: presentations on antivirals, traditional therapies and host-directed interventions at the 5th ISIRV Antiviral Group conference. Antiviral Res 149:118–142. 10.1016/j.antiviral.2017.11.013. 29162476 PMC7133686

[25] Yan D, Lee S, Thakkar VD, Luo M, Moore ML, Plemper RK. 2014. Cross-resistance mechanism of respiratory syncytial virus against structurally diverse entry inhibitors. Proc Natl Acad Sci USA 111:E3441–E3449. 10.1073/pnas.1405198111. 25092342 PMC4143008

[26] Cianci C, Genovesi EV, Lamb L, Medina I, Yang Z, Zadjura L, Yang H, D’Arienzo C, Sin N, Yu KL, Combrink K, Li Z, Colonno R, Meanwell N, Clark J, Krystal M. 2004. Oral efficacy of a respiratory syncytial virus inhibitor in rodent models of infection. Antimicrob Agents Chemother 48:2448–2454. 10.1128/AAC.48.7.2448-2454.2004. 15215093 PMC434195

[27] Song JG, Su JC, Song QY, Huang RL, Tang W, Hu LJ, Huang XJ, Jiang RW, Li YL, Ye WC, Wang Y. 2019. Cleistocaltones A and B, antiviral phloroglucinol-terpenoid adducts from *Cleistocalyx operculatus*. Org Lett 21:9579–9583. 10.1021/acs.orglett.9b03743. 31755722

[28] Su JC, Wang S, Cheng W, Huang XJ, Li MM, Jiang RW, Li YL, Wang L, Ye WC, Wang Y. 2018. Phloroglucinol derivatives with unusual skeletons from *Cleistocalyx operculatus* and their *in vitro* antiviral activity. J Org Chem 83:8522–8532. 10.1021/acs.joc.8b01050. 29963868

[29] Tang W, Li MM, Liu YJ, Liang N, Yang Z, Zhao YX, Wu S, Lu SW, Li YL, Liu FY. 2019. Small molecule inhibits respiratory syncytial virus entry and infection by blocking the interaction of the viral fusion protein with the cell membrane. FASEB J 33:4287–4299. 10.1096/fj.201800579R. 30571312 PMC6404555

[30] Wang ZQ, Song QY, Su JC, Tang W, Song JG, Huang XJ, An J, Li YL, Ye WC, Wang Y. 2020. Caffeic acid oligomers from *Mesona chinensis* and their *in vitro* antiviral activities. Fitoterapia 144:104603. 10.1016/j.fitote.2020.104603. 32360288

[31] Samuel D, Xing WM, Niedziela-Majka A, Wong JS, Hung M, Brendza KM, Perron M, Jordan R, Sperandio D, Liu XH, Mackman R, Sakowicz R. 2015. GS-5806 inhibits pre- to postfusion conformational changes of the respiratory syncytial virus fusion protein. Antimicrob Agents Chemother 59:7109–7112. 10.1128/AAC.00761-15. 26324264 PMC4604407

[32] Cianci C, Yu KL, Combrink K, Sin N, Pearce B, Wang A, Civiello R, Voss S, Luo G, Kadow K, Genovesi EV, Venables B, Gulgeze H, Trehan A, James J, Lamb L, Medina I, Roach J, Yang Z, Zadjura L, Colonno R, Clark J, Meanwell N, Krystal M. 2004. Orally active fusion inhibitor of respiratory syncytial virus. Antimicrob Agents Chemother 48:413–422. 10.1128/AAC.48.2.413-422.2004. 14742189 PMC321540

